# Role of Leptin and Adiponectin in Endometrial Cancer

**DOI:** 10.3390/ijms23105307

**Published:** 2022-05-10

**Authors:** Aneta Słabuszewska-Jóźwiak, Aron Lukaszuk, Marta Janicka-Kośnik, Artur Wdowiak, Grzegorz Jakiel

**Affiliations:** 1 First Department of Obstetrics and Gynecology, Centre of Postgraduate Medical Education, Żelazna 90 Street, 01-004 Warsaw, Poland; grzegorz.jakiel1@o2.pl; 2 Saint Sophia Hospital, Żelazna 90 Street, 01-004 Warsaw, Poland; aron@gumed.edu.pl (A.L.); lek.martajanicka@gmail.com (M.J.-K.); 3 Invicta Research and Development Center, Polna 64 Street, 81-710 Sopot, Poland; 4 Chair of Obstetrics and Gynecology, Faculty of Health Sciences, Medical University of Lublin, 4-6 Staszica St., 20-081 Lublin, Poland; wdowiakartur@gmail.com

**Keywords:** leptin, adiponectin, endometrial cancer, signalling pathway

## Abstract

Endometrial cancer is the most common malignancy of the female genital tract. Obesity is a strong risk factor for endometrial cancer. Adipose tissue is an active endocrine organ that synthesizes biologically active cytokine peptides, called adipokines. Adiponectin and leptin are the main cytokines of adipose tissue, which may influence the development of metabolic diseases and carcinogenesis. In this scenario, we describe the role of leptin and adiponectin in the development of endometrial cancer. A better understanding of the signalling pathway of these cytokines in endometrial cancerogenesis will provide an opportunity for effective target therapy and may be usable in fertility-sparing treatment. In the future, clinical trials focusing on adipokines, molecular biology, and genetics of the tumour will be needed.

## 1. Introduction

According to world statistics, endometrial cancer is the second most frequently detected malignant neoplasm of the female genital tract after cervical cancer [1]. It mainly occurs in women over the age of 50 [2], and is associated with obesity, diabetes, and metabolic syndrome. Based on clinical, endocrine, and histopathological features, two types of endometrial cancer are distinguished.

Type I occurs in 80% of cases, includes the histological type of endometrioid carcinoma, and is formed on endometrial hyperplasia, with the presence of atypia, as a result of estrogenic overstimulation. Type II is hormone-independent and includes serous, clear-cell and poorly differentiated carcinomas. It mainly occurs in women in the 7th and 8th decade of life, and is characterized by dynamic growth and poor prognosis [3].

Multiple genetic disorders were found in endometrial neoplasms. Mutations of PTEN, K-ras, β-catenin, and PIK3CA, as well as mutations in mismatch genes (MMR) and microsatellite instability (MSI), are common in endometrial cancer. Type II tumours are dominated by mutations within the p53 and p16 genes, and overexpression or amplification of the HER-2/neu gene. The Genome Atlas project identified four molecular subtypes of endometrial carcinomas: POLE ultramutated with a mutation in the POLE gene, MSI hypermutated with microsatellite instability MSI-H, subtype with a small number of copy alterations—SNCA without genetic markers, and subtype with a high number of copy alterations—SNCA with the TP53 mutation. These subtypes differ not only in genetic disorders, but also in their clinical course and prognosis. It should be noted that obesity is recognized as an independent risk factor for the development of endometrial cancer, regardless of the histopathological type [4].

Adipose tissue is an active endocrine organ that synthesizes biologically active cytokine peptides, called adipokines, that act within the adipose tissue (auto and paracrine), and on distant organs and tissues. Adiponectin and leptin are the main cytokines of adipose tissue, affecting insulin resistance, lipolysis [5], and inflammatory pathways, which may influence the development of metabolic diseases and carcinogenesis [6]. An increased leptin-to-adiponectin ratio is associated with an increased risk of cancer, including endometrial cancer [7,8,9].

## 2. Leptin and Adiponectin

### 2.1. Leptin

Leptin is a 16 kDa adipocytokine encoded by the LEP gene, mainly produced in white adipose tissue [8]. Similarly to many hormones, it is secreted in a free form, responsible for controlling the mass of adipose tissue in the body, and in a form associated with the protein being its soluble receptor. The complex form of the adipokine determines energy expenditure.

**Figure 1:**
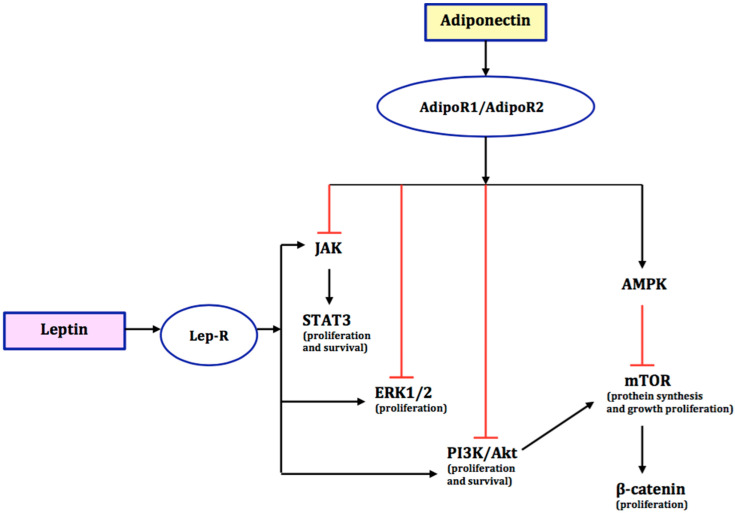
Schematic diagram shows the interaction of leptin and adiponectin and their downstream signalling pathways in endometrial cancer. The black arrows indicate stimulatory effects. The red blunt arrows indicate inhibitory effects. AMPK-AMP—activated protein kinase; ERK1/2—extracellular regulated kinase 1 or 2; JAK—Janus kinase; mTOR—mammalian target of rapamycin; P13K/Akt—phosphatidylinositol 3-kinase/protein kinase B; STAT3—signal transducer and activator of transcription; Lep-R—leptin receptor; AdipoR1/AdipoR2—adipokine receptors.

The structure of leptin is a quadruple helix, very similar to proteins from the cytokine family, such as IL-2, IL-6, IL-12, IL-15, IF, and G-CSF. By acting directly on the nervous system, leptin regulates food intake, and through its peripheral action, it inhibits insulin secretion from pancreatic β-cells stimulated by glucagon-like peptide-1 (GLP-1). In addition, it stimulates lipolysis, glucose utilization, and carbohydrate transport in adipocytes. It also takes part in thermogenesis, haematopoiesis, and osteogenesis, and has pro-angiogenic effects. It has been proven that leptin affects the functioning of the immune system by increasing the pro-inflammatory effects, including the activation of T lymphocytes, increasing the synthesis and release of IL-6 and TNFα, stimulating the response to Th1 lymphocytes, and increasing the activity of NK cells, macrophages, and neutrophils by intensifying their chemotaxis [10]. The action of leptin at the cellular level is related to the presence of membrane receptors (Ob-R), widespread in target organs (brain tissue, heart, lungs, kidneys, liver, pancreas, intestines, placenta, gonads, spleen, and thymus). The membrane receptor is a glycoprotein that is similar in structure to class I cytokine receptors. The gene encoding the leptin receptor is located on the 1p32 chromosome. It is responsible for the synthesis of at least five isoforms: long form—OB-Rb—showing the ability to transmit intracellular signals, three short forms—OB-Ra, OB-Rc, and OB- Rd, and one soluble receptor isoform—OB-Re. Within the OB-Rb receptor, the presence of an extracellular domain, with leptin binding sites, and a transcellular domain, with interaction sites for Janus kinase (JAK) and STAT (signal transducers and activators of transcription) proteins, has been demonstrated. The mechanism of action of leptin is, among others, based on the activation of STAT signalling proteins. Within the OB-Rb receptor, it homodimerizes and activates JAK protein kinases, which are responsible for the phosphorylation of STAT signalling proteins. After phosphorylation, these proteins move to the cell nucleus, where they bind to the appropriate gene, stimulating its transcription. Moreover, leptin stimulates cancer stem cells, which leads to cell proliferation and tumour growth [11,12,13]. There is a positive correlation between leptin levels and body mass index (BMI). Elevated levels of leptin and its receptors may potentially contribute to the progression of cancer [14]. The role of leptin and adiponectin in signalling pathways in carcinogenesis is presented in Figure 1.

### 2.2. Adiponectin

Adiponectin, apM1 (adipose most abundant gene transcript 1), is a peptide hormone that is mainly synthesized in white adipose tissue, which plays an important role in glycaemic and lipidemic homeostasis. Adiponectin is made of 244 amino acids and comes in two forms: homotrimers with a low molecular weight (LMW—low molecular weight), and in the form of high-molecular-weight oligomers (HMW—high molecular weight). This cytokine has an affinity for two receptors—AdipoR1, found mainly in skeletal muscle, and AdipoR2, found in liver tissue [15,16,17]. Both receptors are in the endometrium, in the placenta [18], and in neoplastic tissue, including endometrial cancer [19]. Adiponectin, in contrast to leptin, has antiproliferative, anti-angiogenic and anti-atherosclerotic effects [20]. It has a selective influence on mitogenic growth factors, i.e., fibroblast growth factor, platelet-driven growth factor BB (PDGFBB), fibroblast growth factor (FGF), and heparin-binding epidermal growth factor-like growth factor (HB EGF), which causes a reduction in the bioavailability at the pre-receptor stage, thus weakening the DNA synthesis and cell proliferation induced by these factors [21]. Hence, the decreased levels of adiponectin observed in obese individuals may become a carcinogenic factor.

The major signalling pathway for adiponectin involves AMP-activated protein kinase (AMPK) [22]. AMPK inhibits key kinase signalling pathways, such as extracellular ½-regulated kinase (ERK1/2), phosphatidylinositol-3-kinase (PI3K), protein kinase B (Akt), c-Jun N-terminal kinase (JNK), terminal kinase (cJNK), and STAT3, which are involved in the cell cycle that guarantees proper cell growth and survival [23,24,25,26]. The AMPK pathway is also crucial for cell growth by regulating Akt/mTOR/S6K transmission. It is presented in Figure 1. Thus, decreased AMPK phosphorylation leads to the stimulation of cell proliferation [27], and its activation can inhibit cell growth and initiate apoptosis [28,29]. AMPK can be phosphorylated and activated by LKB1 and by calmodulin-dependent protein kinase kinase-β (CaMKK-β) [30,31,32]. It should be emphasized that LKB1 is a tumour suppressor protein, and is an upstream kinase of tumour energy [33].

Moreover, adiponectin can directly regulate the expression of proteins involved in the cell cycle and apoptosis by affecting p53 and Bax (up-regulating), as well as c-myc, cyclin D1, and Bcl-2 (down-regulating) [34]. This cytokine also has the ability to inhibit the phosphorylation of the nuclear transcription factor κB (NF-κB), thus influencing the activity of various pro-inflammatory mediators [35].

Adiponectin is also involved in the energy metabolism of the organism, intensifying the process of lipid oxidation in peripheral tissues (white adipose tissue and muscle tissue) by stimulating the synthesis and activity of enzymes involved in triglyceride metabolism (Acyl-CoA oxidase, 5-protein kinase, and triglyceride lipase). In addition, it inhibits triglyceride synthesis and gluconeogenesis in the liver, and plays a significant hypoglycaemic and lipid-lowering role [16,36]. By activating the nuclear receptor for PPARγ, which is associated with insulin resistance, adiponectin has become a marker of both insulin sensitivity and insulin resistance.

## 3. Leptin Effect on the Development of Endometrial Cancer

Neoplastic tumour progression is based on the unlimited ability of its cells to proliferate and their potential immortality. The continuous growth and development of the tumour depends on providing it with increased amounts of oxygen and nutrients, and those can only be sustained by neoangiogenesis. At a later stage, epithelial–mesenchymal transformations are initiated, aimed at cancer cells acquiring the ability to move to blood and lymphatic vessels, and then to spread all over the body. The emerging hypoxia in neoplastic tissue regulates the expression of various growth factors and their receptors.

HIF-1 is a marker of oxygen deficiency in any tissue, including neoplastic; hence, the overexpression of this factor in endometrial cancer is associated with stimulated angiogenesis and an unfavourable prognosis [37]. HIF-1 overexpression is associated with increased transcription of the leptin gene and its receptor (ObR) [38,39,40]. Both leptin and HIF-1 activate STAT (signal transducer and activator of transcription) proteins, which are related to the invasiveness of the endometrial cancer cell line [41]. STAT proteins play a role in the transduction of extracellular signals, and belong to the direct transcription factors regulating gene expression [42]. The STAT protein family consists of seven members (STAT1, STAT2, STAT3, STAT4, STAT5a, STAT5b, and STAT6). These proteins are associated with the inflammation, survival, proliferation, metastasis, angiogenesis, and chemoresistance of neoplastic cells [43].

Initially, they are present in the cytoplasm in an inactive form and become activated after binding to signalling peptides, which include cytokines, growth factors, and hormones. When activated by cytokines, the cytokine receptor dimerizes and then binds to the cell membrane, leading to the activation of JAK (Janus-activated kinase) by rapid phosphorylation of cytoplasmic domains. Phosphorylation generates a recognition site for STAT proteins with a homologous Src-binding domain (SH2—Src homology2), allowing them to form homo- or heterodimers and rapidly translocate to the cell nucleus. In the nucleus, dimers bind to specific sequences (often referred to as activated sequences) in gene promoter regions to stimulate their transcription [44]. In endometrial cancer, leptin activates STAT3 proteins, which increase their activity in the process of oncogenesis by stimulating proliferation [45], promoting angiogenesis, and avoiding the control of the immune system. A study on an endometrial cancer cell line showed that activated STAT3 proteins overexpress anti-apoptotic genes, such as Bcl-xL and Mcl-1, rendering these cells immortal and chemo-resistant [46]. In a study on SPEC-2 cell lines, leptin, as opposed to adiponectin, led to an increase in the activity of metalloproteinases MMP-2 and MMP-9, which are associated with the aggressiveness of the neoplastic process. Moreover, a higher tissue concentration of leptin positively correlates with the degree of myometrial invasion and lymph node metastases, eventually leading to a worse prognosis [47]. Similar conclusions were provided by the study by Cymbaluk-Płoska et al., which showed that higher levels of serum leptin correlate with the involvement of lymphatic vessels and with a low degree of neoplastic differentiation [48]. Hence, inhibition of the leptin-promoted STAT3/JAK signalling pathway may limit tumour metastasis.

Notch is an evolutionarily conserved cellular information pathway that influences the differentiation, proliferation, and apoptosis of cells at various stages of their development [49]. Changes in the Notch signal are closely related to the occurrence of genetic and autoimmune diseases, and the development of neoplasms, including breast, pancreatic and endometrial cancer [50,51]. Notch receptors are activated by contact with a ligand (Jagged or Delta) from a neighbouring cell. After ligand binding, the receptor undergoes proteolytic processing, as a result of which the NICD (notch intracellular domain) is detached from it and travels to the cell nucleus, where it acts as a transcription factor. In the nucleus, NICD forms a complex with the DNA binding protein of CSL, displacing the histone deacetylase (HDAc)–corepressor (CoR) complex with CSL. Subsequently, components of the activation complex, such as MAML1 and histone acetyltransferase (HAT), are recruited into the NICD-CSL complex, leading to transcriptional activation of Notch target genes. All four Notch receptors were found to be present in the endometrium. Notch 1 and Notch 3 are mainly located in the glandular epithelium, while Notch 4 is only in the stroma of the endometrium [52]. It should be emphasized that the expression of these signalling pathways changes with the phase of the menstrual cycle [52,53] and decreases after menopause [54]. Many years of research on the activity of individual receptors and ligands of this evolutionarily old signalling pathway in endometrial cancer have provided different, sometimes contradictory, results. However, most authors agree that both mRNA and Notch 4 protein expression are reduced [52,54,55,56,57,58,59,60,61]. The role of leptin in the regulation of Notch pathways has been well established in breast cancer, where, together with IL-1, Notch upregulates ligands, receptors, and relevant genes, thus enhancing the induction of proliferation and migration of cancer cells, as well as chemoresistance [62,63,64,65,66]. In a study on the HEC-1A and Ishikawa cell lines (type I endometrial cancer), and KLE and An3Ca (type II endometrial cancer), the same crosstalk of leptin and IL-1 was associated with greater invasiveness and chemoresistance by inducing all Notch receptors (NOTCH1-4), their ligands, JAG1 and DLL4, and downstream effectors (survivin and HEY2). This mechanism is described in both types of endometrial cancer, although it is stronger in type II, in which leptin increased the proliferation of cancer cells [67]. Thus, leptin becomes the cytokine responsible for the proliferation of endometrial cancer. Therefore, the implementation of a treatment that reduces the blood levels of leptin or inhibits the cascades of signalling dependent on this adipokine may limit the development of neoplastic disease [68].

## 4. The Role of Adiponectin in the Development of Endometrial Cancer

Epidemiological studies confirm the association between low adiponectin levels, obesity, and increased incidence of cancer [69]. The European Prospective Investigation into Cancer and Nutrition (EPIC) study found that lower plasma levels of adiponectin in peri- and postmenopausal women predispose them to an increased risk of developing endometrial cancer, regardless of BMI status and other obesity-related risk factors, such as circulating C-peptide levels, sex hormones, IGF-1 binding protein (IGFBP-1), and peri- and postmenopausal IGFBP-2 [70].

Low levels of adiponectin measured in blood serum (<8 mg/L) are associated with greater aggressiveness of the neoplastic process, more frequent metastases in the lymph nodes, and a low degree of histopathological differentiation [71]. It should be emphasized that an elevated leptin-to-adiponectin ratio is also an independent factor in the development of endometrial cancer [72]. In contrast, high preoperative levels of adiponectin are a positive prognostic factor, in terms of disease-free time and overall survival, in endometrial cancer patients [73].

The suppressor protein PTEN is a lipid phosphatase with dual specificity for both proteins and lipids, being a negative regulator of the PI3K-Akt-mTOR signal axis, thereby controlling cell survival, proliferation, and growth processes. This protein plays a key role in silencing the stimulation transmitted from EGFR, HER-2 and IGFR receptors to the Akt signalling cascade. Somatic mutation of PTEN, leading to complete or partial loss of the PTEN protein, results in the development of a neoplastic process characterized by high dynamics of tumour growth, worse prognosis, and faster clinical manifestation of the proliferative process. A study conducted in an animal model confirmed that a heterozygous PTEN mutation leads to the formation of endometrial cancer in as many as 30% of cases, but this effect may be exacerbated by adiponectin deficiency, due to activation of the mitogen-activated protein kinase (MAPK) pathway [74].

The antiproliferative effect of adiponectin is related to the presence and functionality of AdipoR receptors, as well as the expression of the *LKB1* suppressor gene [32,75]. Studies on endometrial cancer cell lines have shown that knockdown of the *LKB1* gene abolishes adiponectin-induced reductions in cellular activity, including cell proliferation, colony formation, adhesion, and invasion, in endometrial cancer cell lines [76]. LKB1-mediated adiponectin signalling may also interact with the protein PTEN involved in the regulation of the cell cycle. It should be noted that a large proportion of endometrial neoplasms contain mutations in *PTEN*. Moreover, the inhibition of cell cycle regulators, cyclin D1 and E2, as well as ERK1/2 and Akt signalling proteins, by adiponectin confirms its antiproliferative activity [77]. In studies on endometrial cancer cell lines, adiponectin has an inhibitory effect on the signalling pathways stimulated by leptin, which makes its antiproliferative and pro-apoptotic effects stronger [78].

## 5. The Role of Oestrogens in the Development of Endometrial Cancer

Oestrogens play a mitogenic role in the normal endometrium, which consists of the functional and basal layers. The functional layer covers two-thirds of the thickness of the endometrium and peels off at the end of the luteal phase of the menstrual cycle. It separates from the basal layer, which remains intact and forms the basis for endometrial regeneration. The ovaries are the main source of oestrogen, and the levels of oestrogen fluctuate with the phases of the menstrual cycle. In the follicular phase, oestrogens, i.e., estrone and 17β-oestradiol, are produced by the granulosa cells of a growing ovarian follicle and lead to the growth of the endometrium [79], mainly its glands, which are initially thin, tubular and lined with low cells of the cylindrical epithelium. With time, as a result of numerous mitotic divisions, the adjacent glands fuse with each other. The highest levels of oestradiol are reached at the end of the follicular phase, and then decline sharply after ovulation. This is followed by a re-rise in oestrogen levels in the middle luteal phase with a decline towards the end of the menstrual cycle.

In the following days, oestrogens are produced by the corpus luteum formed after ovulation, but their levels are already lower and drop significantly just before menstruation [80,81]. In the second phase of the cycle, because of the action of progesterone produced by the corpus luteum, the growth of the endometrium is inhibited, which is associated with a decrease in mitotic activity and DNA synthesis. Progesterone also interferes with the expression of oestrogen receptors, and stimulates the activity of 17β-hydroxysteroid dehydrogenase and sulfotransferases, which convert oestradiol into estrone sulphate, which is quickly excreted from the cell. An imbalance between the oestrogens that lead to the growth of the endometrium and the progesterone that inhibits them can lead to endometrial hyperplasia and endometrial cancer. In menstruating women, the ovaries are the main source of oestrogen, and after menopause, these hormones mainly come from the extra-glandular conversion of androstenedione and testostosterone.

Adipose tissue is a source of enzymes, such as cytochrome P450-dependent aromatase, 11β-hydroxysteroid dehydrogenase type 1 (11βHSD 1), and 17β-hydroxysteroid dehydrogenase (17β-HSD), involved in the synthesis and metabolism of steroid hormones [82]. Cytochrome P450 and 17β-HSD-dependent aromatase are highly active in adipose tissue stromal cells and pre-adipocytes. Cytochrome P450-dependent aromatase catalyses the conversion of androstenedione to estrone, and testosterone to oestradiol, while 17-βHSD is involved in the conversion of androstenedione to testosterone, and estrone to oestradiol [83]. The 17-βHSD aromatase enzyme activity ratio positively correlates with abdominal obesity. The percentage of androstenedione converted to oestrogens positively correlates with body weight. Most of the major sex steroids, such as oestradiol and testosterone, circulate in the blood in the form of a protein carrier, sex hormone-binding globulin (SHBG). In obese women, the reduction in SHBG protein leads to an increase in the concentration of free oestrogens, contributing to the development of endometrial hyperplasia and cancer. Thus, obese women have a three times higher risk of developing endometrial cancer [84].

Free oestrogens are active hormones that bind to ERα or ERβ receptors to produce a biological effect [85]. These genomic receptors belong to the nuclear receptor family and act as transcription factors, or interact with other transcription factors, such as Sp-1, AP-1 and NF-κB, to express genes related to cell proliferation, differentiation, and normal function [86,87]. Oestrogen receptors show activity dependent on the binding of the oestrogen ligand, increased amounts of which contribute to the development of endometrial hyperplasia or cancer [88]. Endometrial cancer has been divided into oestrogen-dependent type I, which accounts for approximately 80% of all ERα-predominant cases, and into poorly differentiated and more aggressive type II. Although, for many years, it was believed that type II is oestrogen independent [89,90], more and more experimental studies prove the lack of differences in the expression of oestrogen receptors and the levels of sex hormones between these types of neoplasms, which would suggest estrogenic aetiology in both cases [91,92].

The results of studies on the expression and role of ERβ in endometrial cancer are ambiguous. Studies conducted on animal and human models indicate the suppressive role of ERβ in the development of endometrial cancer [93,94,95,96]. However, an elevated, altered ERβ, ERβ5 isoform is found in endometrial cancer, which is associated with the expression of HER 2 and MyBL2 oncogenes [97,98]. The analysis of endometrial cancer data from the Genome Atlas showed that the mean expression level of ERα was 2.9 times higher than ERβ [99], which may be due to the higher levels of gonadotrophins in the postmenopausal period. Since ERβ acts as a negative regulator of ERα, postmenopausal low expression of ERβ may promote the proliferative effects of ERα [93].

ERα and ERβ are highly homologous in their DNA-binding domains (DBDs) and possess moderate sequence identity in their ligand-binding domains (LBDs). Within the oestrogen receptor, there are six regions located in five domains. In the regulatory domain, there is an A/B region whose function is to activate TAF-1 transcription. TAF-1 can stimulate transcription without binding to the hormone. The C region, located in the DNA-binding domain, is essential for the activation of transcription. The D region is located between the DNA-binding domain and the hormone-binding domain, and plays an important role in the transport of the cytoplasm-synthesized receptor into the cell nucleus. The E region is in the hormone-binding domain and is responsible for dimerization and the activation of TAF-2 transcription. In addition, it is a binding site for heat shock proteins (hsp90), which may interfere with the membrane transport of the receptor and the dimerization process of DNA binding. The F region modulates gene transcription and influences its conformation. Both ERα and ERβ have nearly identical DNA-binding domains, but differ in the ligand-binding domain [86].

**Figure 2:**
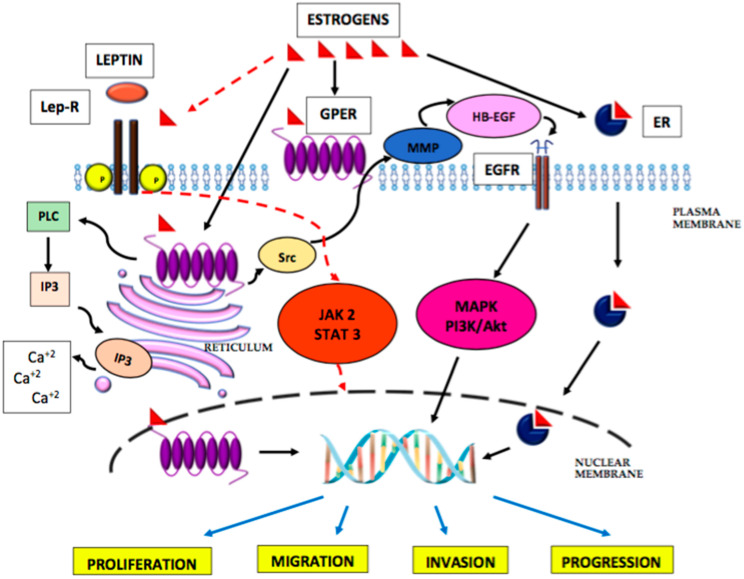
Interaction of oestrogens and leptin and their downstream signalling pathways in endometrial cancer. JAK—Janus kinase; STAT3—signal transducer and activator of transcription; P13K/Akt—phosphatidylinositol 3-kinase/protein kinase B; Lep-R—leptin receptor; ER—oestrogen receptors; GPER—G-protein-blinded oestrogen receptor; EGFR—epidermal growth factor receptor; MMP—metalloproteinase; P—phosphorylation; IP3—inositol 1,4,5-trisphosphate; PLC—phospholipase C; Src—protein tyrosine kinase.

The transcriptional activity of oestrogen receptors may be increased by phosphorylation derived from the cAMP and kinase A pathways. Thus, growth factors, such as epidermal growth factor-EGF, IGF-I and transforming growth factor alpha (TGFα), by stimulating phosphorylation of the kinase, may activate oestrogen receptors in the absence of oestrogen. In addition to nuclear receptors ERα and ERβ, a G-protein-blinded oestrogen receptor, called GPER or GPR 30, has been identified, which is present within the cell membrane, endoplasmic reticulum and in the cell nucleus [100]. This receptor has a high affinity for oestradiol and a low affinity for estrone and estriol [87,101], with estrone and oestradiol being its agonists and estriol being its antagonist. The GPER receptor mediates non-genomic oestrogen signalling via cAMP or via calcium ions released through it from the endoplasmic reticulum by PLC. By activating the Src protein, GPER promotes the activation of metalloproteinases 2/9, causing EGFR transcription, and by stimulating the MAPK and PI3K/Akt signalling pathways, it can influence the cell cycle, proliferation, differentiation, apoptosis, migration, and cell invasion. Hence, activation of this receptor may modulate carcinogenesis. Endometrial cancer studies have shown GPER overexpression, which correlated with the aggressiveness and advancement of the neoplastic process, as well as a worse prognosis, regardless of the menopausal status. In the case of non-endometrioid types of tumours, a loss of GPER expression was observed, as compared to the normal endometrium, which correlated with the advancement of the disease, low histopathological differentiation, and low expression of the ERα receptor. However, there are studies showing no differences in GPER expression between endometrial cancer types I and II [102]. It should be noted that in endometrial cancer cells, the expression of GPER, as opposed to ERα [103], is three times lower compared to the normal endometrium. The signalling pathway of oestrogens in carcinogenesis is presented in Figure 2.

Epidemiological studies have shown obesity to be a major risk factor for the development of endometrial cancer in postmenopausal women [104], as well as being the main source of oestrogen and adipokines during this period of life. Studies carried out on an animal model confirm the mutual synergy of action between oestradiol and leptin in the aetiology of endometrial cancer development, which is manifested by transactivation of the Ob-R receptor through mutual activation of the JAK-STAT pathway [105], and this is presented in Figure 2. Thus, oestradiol, directly on the endometrium and indirectly increasing the effect of leptin, may contribute to the development of endometrial cancer. However, this hypothesis requires further clinical research.

## 6. Summary

Many epidemiological studies indicate obesity as an independent risk factor for endometrial cancer in postmenopausal women. Moreover, endometrial cancer appears in about 4% of women at reproductive age [2], which can create difficulties in identifying adequate treatment and fertility preservation [106], which is why these patients very often need multidisciplinary care, such as oncological and psychological [107]. A better understanding of molecular biology, particularly regarding the genetics of tumours, can improve the selection of patients who may be eligible for fertility-sparing treatment [108,109]. Further studies of leptin and adiponectin signalling pathways and their “cross talk” with oestradiol may allow for the development of an effective targeted therapy in endometrial cancer.
